# Consumption of Hydrogen by Annihilation Reactions in Ultradense Hydrogen H(0) Contributed to Form a Hot and Dry Venus

**DOI:** 10.1089/ast.2022.0131

**Published:** 2023-10-17

**Authors:** Leif Holmlid, Frans Olofson, Dan Gall

**Affiliations:** ^1^ Department of Chemistry and Molecular Biology, University of Gothenburg, Göteborg, Sweden.; ^2^ LazeraH AB, Göteborg, Sweden.

**Keywords:** Annihilation, Ultradense hydrogen, Water depletion, Gamma radiation

## Abstract

When water vapor reacts with metals at temperatures of a few hundred kelvin, free hydrogen and metal oxides are formed. Iron is a common metal giving such reactions. Iron oxide together with a small amount of alkali metal as promoter is a good catalyst for forming ultradense hydrogen H(0) from the released hydrogen. Ultradense hydrogen is the densest form of condensed matter hydrogen. It can be formed easily at low pressure and is the densest material in the Solar System. Spontaneous and induced nuclear processes in H(0) create mesons (kaons, pions) in proton annihilation reactions. It is here agreed on that the great difference in the present conditions on Venus and Earth are caused by the initial difference in the temperatures of the planets due to their different distances from the Sun. This temperature difference means that, in warmer planetary environments such as on Venus, the iron + water steam → iron oxide + hydrogen reaction proceeded easily, meaning a consumption of water to give H(0) formation and release of nuclear energy by subsequent nuclear reactions in H(0). On the slightly cooler Earth, the iron + liquid water reaction was slower, and less water formed H(0). Thus, the water consumption and the heating due to nuclear reactions was smaller on Earth. The experiments proving that the mechanisms of forming H(0) and the details of the nuclear processes have been published. The more intense particle radiation from the nuclear processes in H(0) and the lack of water probably impeded formation of complex molecules and, thus, of life on planets like Venus. These processes in H(0) may, therefore, also imply a narrower zone of life in a planetary system than believed previously.

## 1. Introduction

Baryon annihilation reactions in ultradense hydrogen H(0) have been studied in detail (Holmlid, 2021, 2022a; Holmlid and Olafsson, 2021). H(0) is one type of hydrogen condensed matter that is related to metallic hydrogen and Rydberg matter (see, *e.g.,*Holmlid and Zeiner-Gundersen, 2019). There are 65 published papers on this type of matter. H(0) is the thermally most stable material (bond energy >500 eV) in the universe (Holmlid, 2019). It is also chemically quite inert. H(0) will exist everywhere where hydrogen exists, as in the interstellar medium and in most stars (Holmlid, 2017a). H(0) forms very small strongly bound molecules of different shapes (Holmlid and Zeiner-Gundersen, 2019). The most common shape is the chain-molecule H_2*N*_(0), with typical molecular sizes of 5–50 pm and interatomic distances of 2.245 pm in the most easily formed spin state *s* = 2. This small size means that H(0) molecules can exist interstitially particularly in metals and metal oxides and be observed only as dissolved hydrogen.

Due to the very short internuclear distances in H(0), nuclear processes can easily be induced in this material. The main type of nuclear reaction is annihilation, which is easily observed by the high energy release, a factor of >100 more energy than from fusion (Holmlid, 2022b). The annihilation reactions in H(0) consume hydrogen and release large amounts of energy, mesons and gamma radiation (Holmlid, 2022a). When the consumed hydrogen originates in water, this process may help explain the difference between the planets Venus and Earth, giving a drier and much hotter Venus. The proposed mechanism is not in conflict with other more general and established processes involved in the development of planets like Venus and Earth (Hunten *et al.,* 1983; Taylor, 2014; Lammer *et al.,* 2018, 2020, 2021) but is proposed to be a decisive contributing factor to the strongly different developments of the planets.

When water, particularly steam, reacts with a transition metal like iron at relatively high temperatures, various forms of metal oxides and free hydrogen are formed. The metal oxide together with a small amount of alkali salt as promoter is a superior catalyst for forming ultradense hydrogen H(0) from the released hydrogen (Holmlid *et al.,* 2021). These processes have been studied in the adsorbed phase on the iron oxide surface. There is no indication that they would be faster in a condensed phase. Baryon annihilation processes in H(0) create mesons (kaons, pions) (Holmlid, 2021, 2022a). The kaons and pions decay rapidly to muons and finally to gamma and beta radiation. Hence, hydrogen is transformed by the annihilation reactions to mesons and high-energy radiation and finally to heat. Of course, this particle and gamma radiation will tend to destroy any organic molecules formed. In this way, any form of biological development will be strongly impeded on a planet like Venus or any other planet where H(0) annihilation reactions are active.

This H(0) baryon annihilation mechanism, which has been thoroughly studied in the laboratory (Holmlid 2021, 2022a; Holmlid and Olafsson, 2021), means water depletion, increased temperature, and impeded life development. This agrees with what we see today on Venus, contrary to Earth. The main reason for the low rate of annihilation reactions on Earth is the longer distance from the Sun that allows water condensation and thus leads to much slower water-metal reactions.

It is agreed that the great difference in the present conditions on Venus and Earth is caused by the difference in the initial temperatures of the planets due to their different distances from the Sun. This means that, on the warmer Venus, the metal + water steam reaction produced metal oxide + hydrogen, meaning a consumption of water to then give H(0) formation and release of nuclear energy by subsequent nuclear reactions. On the cooler Earth, with liquid water, the water + iron reaction was different, producing a catalytically less efficient metal oxide. Thus, less hydrogen formed H(0), and the water consumption and the nuclear heating were smaller. Other mechanisms amplified this difference. For example, an elevated temperature of the oxide means that its alkali promoter can function more efficiently to produce H(0). Increased temperature means increased mobility and increased rate of desorption for the alkali promoter atoms. The mechanisms of forming H(0) and the details of the nuclear processes have been published previously, and they are cited above.

## 2. Properties of Hydrogen Condensed Matter H(0), H(1), and H(*l*)

**FIG. 1.**
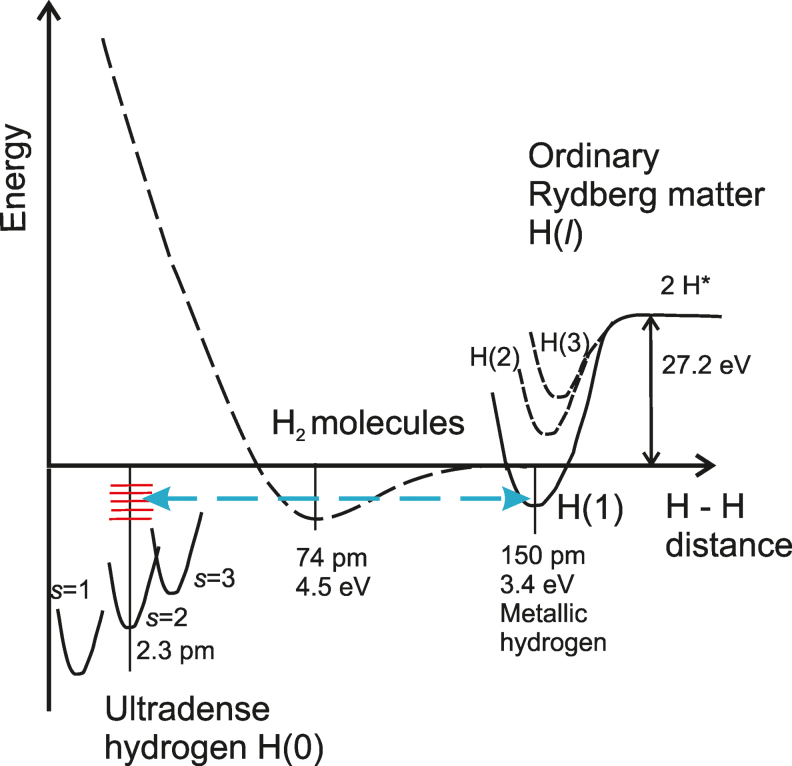
Relations between the three main types of hydrogen condensed matter H(0), H(1), and H(*l*). Not to scale. Covalently bound hydrogen molecules are also included for comparison. Color images are available online.

The properties of hydrogen condensed matter (or hydrogen Rydberg matter) are determined by the orbital angular momentum *l* of its electrons. The three main types differ in this respect. One type has *l* = 0, that is H(0) or ultradense hydrogen (see, *e.g.,* Holmlid and Zeiner-Gundersen, 2019). The second type has *l* = 1, that is H(1) or metallic hydrogen. Finally, general hydrogen Rydberg matter has *l* > 1 (see, *e.g.,* Holmlid [2012] for a previous review). These three types differ by their internuclear distances, which increase by a factor of 137 (1/α inverted fine structure constant) from H(0) to H(1) and from H(1) to H(*l*) (with *l* ≈ 12; see Fig. 1). The basic description of the three levels of matter is adapted from the work of Hirsch (2012). One further quantum number determines the properties of H(0), namely, the spin quantum number *s,* which gives the internuclear distance of H(0). The spin takes values *s* = 1, 2, 3, 4 … (observed in experiments [Holmlid, 2017b, 2018]). The spin gives the internuclear distances in H(0) as *d* = 2.9 × 0.192 × *s*^2^ pm (Holmlid and Zeiner-Gundersen, 2019). This means an internuclear distance of 0.56 pm in state *s* = 1 and 2.245 pm in *s* = 2 (Holmlid, 2018). Spin state *s* = 2 is the easiest spin state to produce in the laboratory.

For spin state *s* = 1, the internuclear distance is so small that nuclear processes are spontaneous at a low rate. These processes create antibaryons and baryon annihilation, mainly proton + antiproton → mesons. The mesons created from the annihilation are charged and neutral pions and charged and neutral kaons. These mesons decay rapidly to form mainly muons and some gamma photons (Particle Data Group, 2022). The spontaneous annihilation rate at room temperature and low external pressure in state *s* = 2 is measured to be approximately10^−25^ s^−1^. Thus, an H atom in H(0) can be assumed to live much longer than the estimated age of the universe before annihilating. This means that primordial clouds of H(0) can still exist in our universe. When a transfer to *s* = 1 is induced by external means like particles and photons, the nuclear processes are expected to start in about 1 ns, as at similar internuclear distances in muon-induced fusion (Balin *et al.,* 2011). The deexication can be induced by laser pulses and has been extensively studied (Holmlid, 2021, 2022a). High-energy particles, particularly charged particles, will also induce annihilation. Intense UV light may have a similar effect.

Ultradense hydrogen has been criticized unscientifically (Hansen and Engelen, 2022a, 2022b) without attempting a repetition of the experiments. Unscientifically means that the results in the 65 publications on H(0) were not disproved but just rejected since they were not understood. To date, no experimental evidence that falsifies the existence or any property of ultradense hydrogen has been published or mentioned. The critical comments noted above have been fully answered by two scientific responses (also on arXiv, Holmlid and Kotzias, 2022; Holmlid, 2023).

## 3. Chemistry on Venus

### 3.1. Chemical processes on early Venus

**FIG. 2.**
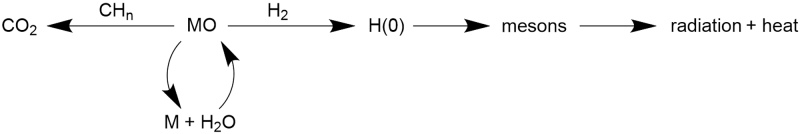
Circular reaction diagram for the processes producing radiation, heat, and CO_2_ from water and hydrocarbons.

A circular reaction diagram for the chemical processes on Venus discussed here is shown in Fig. 2. As is normally done, it is assumed that Venus and its protoplanets contained some water. The view that *condensed* water never existed on the surface of Venus, due to its high temperature, seems to be common (Hamano *et al.,* 2013; Lammer *et al.,* 2018 and references therein). As described below, this type of reaction is expected to produce the metal oxides needed to form H(0) if the metal oxides do not exist previously in the form of magma seas. Lower temperatures and liquid water as on Earth instead tend to form mixed hydroxide-oxides like rust that is not an efficient catalyst for H(0) production.

When water at high temperature reacts with metals like iron, it forms metal oxides and free hydrogen. A temperature above 375 K, thus steam, is needed for rapid reaction. Hydrogen will also exist in the atmospheres of the protoplanets. Thus, a reducing steam atmosphere, such as proposed for early Venus, would drastically increase the formation rate of H(0).

Magma oceans in protoplanets (Elkins-Tanton, 2012; Hamano *et al.,* 2013; Lebrun *et al.,* 2013; Ikoma *et al.,* 2018) that contain metal oxides and alkali metals seem to have been perfect locations where H(0) was formed and annihilation reactions took place. The annihilation reactions would then contribute to the heating of the magma. According to Hamano *et al.* (2013) “The thermal evolution of a magma ocean is closely linked to the formation of a steam atmosphere.” The water content and partioning in protoplanets was also considered by Ikoma and Genda (2006) and Ikoma *et al.* (2018). H(0) forms clouds (Andersson *et al.,* 2011) consisting of small droplets of the various types of H(0) molecules, so H(0) formation and annihilation reactions in an aerosol in the protoplanetary atmospheres are likely.

As an example of the metal oxide formation, the total reactions with iron are

(1) $$2Fe+3H_{2}O\to Fe_{2}O_{3}+3H_{2}$$

and

(2) $$3Fe+4H_{2}O\to Fe_{3}O_{4}+4H_{2}$$

Iron oxide Fe_2_O_3_ is the best catalyst known to form H(0) from hydrogen gas (Holmlid *et al.,* 2021). Alkali metals are used as catalyst promoters (Holmlid *et al.,* 2021). Also, alkali promoted Fe_3_O_4_ is useful for forming alkali Rydberg species and thus H(0) with a lower efficiency as well (Engvall *et al.,* 1999; Holmlid *et al.,* 2021). The most often used alkali metal in the laboratory is K, but other alkali metals like Na and Li are known to function as promoters. Discussions about the fate of potassium in protoplanets have been put forth (see, *e.g.,* O'Neill *et al.,* 2020; Erkaev *et al.,* 2023). Other common transition metals like Ni also form good oxide catalysts for H(0). Such metals exist in the iron planets and their protoplanets given that they are the most stable atomic nuclei that exist.

### 3.2. The atmosphere on Venus

The present atmosphere of Venus is almost free of oxygen and consists of CO_2_ to 96.5%. The water content is low, only 0.02%. The pressure is 9.3 MPa or 90 times higher than that of Earth. Recent discussions of the venusian atmosphere include those of Lammer *et al.* (2018, 2021). The average temperature is 735 K. This high temperature is today attributed to the extreme greenhouse effect of the present very dense carbon dioxide atmosphere. It is here suggested that the early heating of Venus was increased by the nuclear processes in H(0), which started early on surfaces in contact with water vapor or hydrogen gas. It is indeed possible that the CO_2_ atmosphere was released from the magma by the solidification of the planetary surface (Elkins-Tanton, 2012; Hamano *et al.,* 2013; Salvador *et al.,* 2017; Benedikt *et al.,* 2020). Thus, we turn to briefly discuss the formation of carbon dioxide on Venus.

### 3.3. Carbon on Venus

The carbon dioxide in the atmosphere on Venus is suggested to be formed from oxygen in the metal oxides, which partially originate in the metal-water reactions in Eqs. 1 and 2 above and exist in the material of the protoplanet. See also Fig. 2. Carbon in most of its compounds can reduce metal oxides and form CO_2_ and metal, as in ordinary metallurgic processes. The metal can then react with water to form hydrogen and subsequently H(0). This cycle, as shown in Fig. 2, will enhance the depletion of hydrogen on the planet. So the large amount of carbon dioxide on Venus just shows that a chemical process has transformed carbon to its most stable state, namely, carbon dioxide with its standard Gibbs free energy change of formation equal to -394 kJ/mol, presumably from small hydrocarbons. The metal helps transfer oxygen from its bound form in water to its very low free energy state in carbon dioxide. The high CO_2_ and low free oxygen content in the atmosphere agree with this scenario, where the necessary energy (heat) to start the processes can come from the annihilation reactions in H(0).

A comparison with Earth may be of interest here. A large amount of the carbon on Earth was also oxidized to carbon dioxide probably by metal oxides and finally deposited as carbonates in the sea. A considerable fraction apparently remained in the form of hydrocarbons and became the basic atoms of life. This agrees with a slower conversion of water to H(0) and less consumption of hydrogen in annihilation reactions. This left much water to form the seas and preserved some carbon in a non-oxidized form, both factors important for the development of life.

The heating of protoplanets and planets by radioactive metals like U, Th, and ^40^K is probably much smaller than from H(0). The energy released by each reacting nucleus is vastly different. The heat budget seems to be an important point for the development of the planets (O'Neill *et al.,* 2020; Erkaev *et al.,* 2023), and baryon annihilation provides much more heat than possible from radioactive nuclei. A ^238^U nucleus with a half-life of 4 × 10^9^ y gives off 4 MeV in an alpha decay. ^40^K with a half-life of 1.2 × 10^9^ y releases 1.5 MeV. A proton in H(0) releases 938 MeV by annihilation, thus a factor of >10 000 more per atomic mass unit. The number of hydrogen atoms in the protoplanets was vastly greater than that of uranium, thorium, and potassium. The annihilation rate may certainly vary strongly due to the conditions. Protoplanets with magma seas seem to be well suited to produce H(0) and thus annihilation heat. It is possible that UV or EUV radiation from the Sun is an efficient agent to induce the annihilation reactions in H(0). The very high corona temperature of the Sun has earlier (Holmlid, 2017a) been proposed to be caused by the nuclear reactions in H(0). H(0) in the Sun is observed from the solar wind proton velocities. At the time of the corona suggestion (2017), the evidence for the annihilation reactions was still only accumulating; thus the broader phrase “nuclear reactions” was used.

### 3.4. Radiation on Venus

The early Soviet probes to Venus—Venera 8, 9, and 10—landed gamma detectors on three different locations on Venus. Venera 8 had the highest-resolution gamma spectrometer and measured unexpected high gamma radiation levels. Venera 9 and 10 measured only in three selected energy regions around the energies from ^40^K, U, and Th (Surkov, 1977). The radiation levels measured by Venera 9 and 10 were lower than those measured by Venera 8. The gamma intensity from the possible basalt rocks on Venus was high but not generally as high as from granite on Earth. The reason for the high gamma intensity measured by Venera 8 is unknown and may, of course, have been due to local variations. Whether the gamma radiation was higher also outside the selected gamma energy regions for Venera 9 and 10 is not known. No gamma spectrometer region covered the positron annihilation gamma energy at 511 keV. This energy is of interest for the baryon annihilation reaction model described here given that decaying positive muons create positrons.

The Pioneer Venus mission from 1978 (Lorenz and Lawrence, 2015) performed gamma measurements in orbit around Venus. It was concluded that gamma radiation from the surface of Venus was unable to penetrate through the thick atmosphere. Only short gamma pulses with a length in the millisecond range were observed and concluded to have originated from atmospheric lightning on Venus and on Earth.

### 3.5. D/H ratio

Venus has a high D/H ratio of 1.9 × 10^−2^ in water in its atmosphere (Donahue *et al.,* 1982), 120 times higher than that of Earth (D/H ratio of 1.6 × 10^−4^). This D/H ratio on Venus is higher than in any other place in the Solar System (Robert *et al.,* 2000). The amount of D on a planet may be due to a complex mix of processes (Pahlevan *et al.,* 2019), but the high value on Venus is difficult to understand from bulk processes. Thus, it is likely due to local nuclear processes. A depletion of p relative to D could be possible by massive annihilation reactions in H(0), but D can also take part in annihilation reactions, so this is not the likely reason for the high D/H ratio on Venus. Instead, processes that directly produce D are much more likely to give the high D/H ratio. The muons produced by the annihilation reactions in H(0) can be captured in many nuclei that become unstable. These unstable nuclei can produce deuterium in subsequent nuclear decays. Results exist on iron group metals, for example ^58^Ni (Heusser and Kirsten, 1972; Measday, 2001). This is the most probable type of process for increasing the D/H ratio on Venus.

## 4. Conclusions

The circular metal-metal oxide reactions shown in Fig. 2 convert water and hydrocarbons to carbon dioxide and ultradense hydrogen H(0). The baryon annihilation reactions in H(0) then destroy the hydrogen and create heat and radiation. This reaction process can help explain the loss of hydrogen and water on Venus, the high temperature on Venus, the formation of the carbon dioxide atmosphere, the high D/H ratio via muon capture and the large gamma radiation on the venusian surface. It is a contributing factor to the very complex processes taking place in the development of terrestrial planets. Since experiments on a large scale are difficult to perform, it is important that all relevant processes are included in the modeling and observation studies.

It would be very interesting to measure the gamma level and the complete gamma spectra on Venus and in particular the positron annihilation gamma radiation at 511 keV to compare it to conditions on Earth. This could show whether the H(0) annihilation reactions are still active on Venus and contribute to the heating of the planet. Recent probes like Messenger and BepiColombo may have found (as yet unpublished) results of gamma radiation at Venus. It would also be useful to measure the muon energy spectrum with converters on photomultiplier detectors (Holmlid and Olafsson, 2015).

## Notes

Associate Editor: Christopher McKay
